# NCs-Delivered Pesticides: A Promising Candidate in Smart Agriculture

**DOI:** 10.3390/ijms222313043

**Published:** 2021-12-02

**Authors:** Qiuli Hou, Hanqiao Zhang, Lixia Bao, Zeyu Song, Changpeng Liu, Zhenqi Jiang, Yang Zheng

**Affiliations:** 1 College of Horticulture and Plant Protection, Yangzhou University, Yangzhou 225009, China; houql@yzu.edu.cn (Q.H.); zhanghanqiao1998@126.com (H.Z.); 007176@yzu.edu.cn (C.L.); 2 Analysis & Testing Center, Institute of Engineering Medicine, Beijing Institute of Technology, Beijing 100081, China; baolixia0228@126.com (L.B.); 3220181090@bit.edu.cn (Z.S.)

**Keywords:** NCs-based pesticides, delivery system, controlled release, pest control, sustainable and smart agriculture

## Abstract

Pesticides have been used extensively in the field of plant protection to maximize crop yields. However, the long-term, unmanaged application of pesticides has posed severe challenges such as pesticide resistance, environmental contamination, risk in human health, soil degradation, and other important global issues. Recently, the combination of nanotechnology with plant protection strategies has offered new perspectives to mitigate these global issues, which has promoted a rapid development of NCs-based pesticides. Unlike certain conventional pesticides that have been applied inefficiently and lacked targeted control, pesticides delivered by nanocarriers (NCs) have optimized formulations, controlled release rate, and minimized or site-specific application. They are receiving increasing attention and are considered as an important part in sustainable and smart agriculture. This review discussed the limitation of traditional pesticides or conventional application mode, focused on the sustainable features of NCs-based pesticides such as improved formulation, enhanced stability under harsh condition, and controlled release/degradation. The perspectives of NCs-based pesticides and their risk assessment were also suggested in this view for a better use of NCs-based pesticides to facilitate sustainable, smart agriculture in the future.

## 1. Introduction

**Figure 1:**
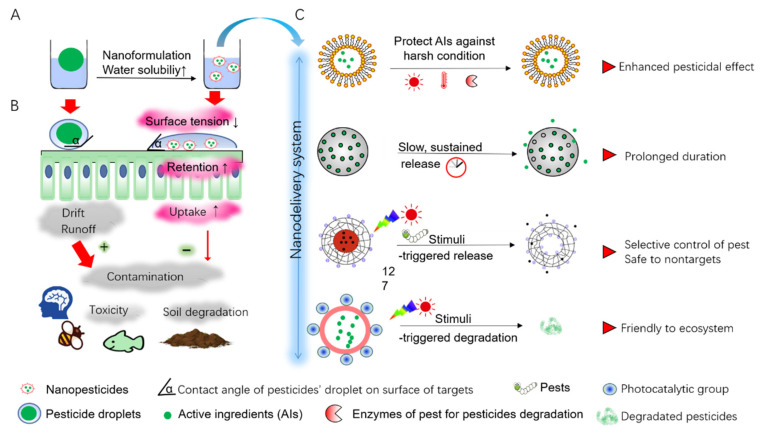
The benefits of nanotechnology for pest control. (**A**) Nanoformulation increase water solubility and stability of pesticidal active ingredients (AIs) by encapsulating AIs into small NCs-based pesticides that are conferring excellent characteristics at nanoscale. (**B**) Nanoformulation reduces surface tension of pesticidal droplets by decreasing the contact angle of droplets on the surface of targets (crop foliage), which contributes to a prolonged retention of droplets and a subsequently increase uptake ratio by target cells. This will be conducive to reduce the contamination caused by pesticides drift or runoff, bringing less toxicity to nontargets (mammal, pollinator, and aquatic organism) and bypassing yield reduction arising from the soil degradation. (**C**) Various nanodelivery systems that play a role in sustainable and smart application of pesticides.

To feed nearly 10 billion people by 2050, food production would need to increase at least 50% from the 2012 level [1]. Chemical pesticides, as one of the most favorable ways for plant protection, have been there used extensively to reduce productive loss caused by pests [2]. However, the excessive use of pesticides has introduced pests to evolve a severe resistance against multiple pesticides. In 2010, more than 550 species of arthropods have developed resistance to at least one insecticide and such resistance has been increasing at an alarming, exponential rate. Fighting with resistant pests has caused a loss of billions of U.S. dollars per year [3]. Unfortunately, resistance is not the only problem caused by unmanaged application of pesticides. The current application mode of insecticide as well as other pesticides such as herbicides, fungicide, and bactericide are relatively extensive and inefficient. Spray, the commonly administrated mode, are typically lost 70% during deposition, partially due to the too large size of pesticidal droplets that inhibit their adhesion to the hydrophobic surface of targets (e.g., the surface of plant foliage or pest cuticle) (Figure 1A) [4,5]. Actually, less than 0.1% pesticides can target pests, resulting in massive pesticide residues in environment [5,6]. These pesticide residues finally become sources of contamination to air, water, and soil, in the form of dust drift, runoff, and rain leaching [5,7,8]. The chemical pollution cycling through ecosystem led to numerous side effects, including soil degradation, lack of productive land, habitat destruction, loss of biodiversity (e.g., aquatic organisms) and elimination of key species (e.g., honey bees) and even other unpredictable hazard effects [9,10,11] (Figure 1A,B). Therefore, extensive attention has been paid to a sustainable application of pesticides that are characterized by enhanced efficacy to targeted pests and reduced side effects to environment.

The long-term unmanaged use of pesticides might be reversed by a new-emerging agri-tech revolution called ‘smart agriculture’ that is based on nanotechnology and aimed at providing agricultural supply for food and industry in an efficient and sustainable manner Equipped with a comprehensive farming system that covers multiple, continuously updated technologies, the ‘smart plant’ in smart agriculture can be edited to report its real-time requirements [12]. Subsequently, the corresponding farming measures of watering, fertilizing or protection are taken automatically by smart pre-programmed machinery systems. This intelligent mode of faming calls for a development of smart pesticides that are able to recognize pests accurately and self-management for release or degradation, thereby enhancing pesticidal efficacy while reducing environmental pollution and other side effects [13]. In recent years, the combination of nanotechnology with strategies in plant protection field have created marvelous smart application in improving pesticides, by conferring conventional pesticides with capability of long duration, regulated performance for release or degradation, and selective control of pests. In this review, we discussed these promising applications and proposed their challenges.

## 2. The Promising Applications of Nanocarriers in Pest Control 

### 2.1. Nanoformulation for Increased Water-Solubility

Most current active ingredients (AIs) of pesticides are commonly hydrophobic compounds with poor water solubility, resulting in the excessive use of both AIs and harmful organic solvents to ensure their dispersion in formulations and subsequent pesticidal effect [14]. Nanotechnology offers new strategy for a good dispersibility of insoluble AIs through an optimized formulation. Nanocarriers (NCs) produced by nanotechnology, with smaller size at nanoscale and various functional groups, can interact with AIs and encapsulate them into multiple nanoformulations such as nanosphere, nanocapsules, nanogels, suspension, and micell, etc. [15,16,17,18,19]. In these nanoformulations, AIs that are poorly soluble or easy to aggregate can be dispersed and encapsulated into the cavities of drug loading matrix composed of NCs, through the chemical interactions between NCs and AIs at proper binding strength. Thus, AIs are conferring with unique properties at nanoscale such as size-dependent qualities and high surface/volume ratio [13]. Compared with conventional formulations, nanoformulations produce smaller pesticidal droplets during application, subsequently improves their adhesive property and retention time, by a reduced surface tension of droplets and decreased contact angle of the droplet on hydrophobic surface of targets (for example, the surface of leaf or insect cuticle) [20]. Also, nanoformulations are responsible for a reduction of toxicity to environment by minimizing organic additives to dissolve AIs [21,22,23], which could help to alleviate various challenges arising from agrochemical contaminations. 

### 2.2. NC-Based Delivery System to Protect Specific AIs against Harsh Condition 

Apart from formulation optimization, targeted application of pesticides is also smart agriculture to ensure pest control effect and reduce toxicity to nontargets. RNA interference (RNAi) mediated pest control has long been a leading part of targeted application of pesticides [24,25,26]. The pesticidal AIs of this strategy is a double strand RNA (dsRNA) that are artificially customized to mimic pest-host RNA viruses. dsRNA can therefore mislead insect pests to trigger the antiviral immune RNAi response that leads to degradation of a specific endogenous gene of pests [27]. Based on this mechanism, a rigorously designed dsRNA can target any pests and any genes except for beneficial organisms theoretically. Moreover, dsRNA is also environmentally benign product, due to its biodegradability in the field. Its promising application has been proposed by thousands of documents, providing hundreds of lethal genes of pests in databases for future insecticidal sources [28,29]. However, the commercialization is still poor. Major obstacles come from inherent limitations of dsRNA. As a biological molecular, it is easy to be degraded under surrounding harsh environmental condition (e.g., Ultra Violet (UV), high temperature). After being blocked by biological barriers and eliminated by endogenous dsRNase of pests, the lethal dsRNA that is identified in screening experiments often displays a limited control effect or shorter duration in field application [28].These obstacles could be downscaled by introducing NCs-based dsRNA delivery system that can not only protect dsRNA against degradation but also help them penetrate the biological barriers of pests [24]. The nanoparticles or NCs can encapsulate dsRNA into dsRNA/NCs complexes that are easy to penetrate biological barriers (e.g., pest cuticles, cell membranes). After entering cells, NCs help dsRNA escape from lysosomal degradation and then target mRNA for an efficient silencing of targeted genes [30]. With development of nanotechnology, multiple NCs (such as chitosan, liposomes, QD and dendrimers, etc.) have been developed and applied in these delivery systems to enhance the pesticidal efficacy [30,31,32]. 

**Figure 2:**
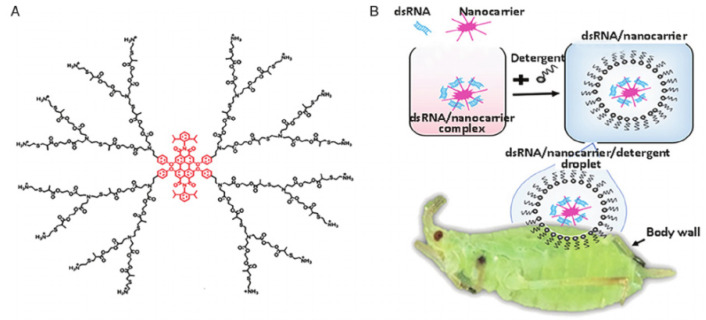
Schematic diagram of NCs-mediated delivery of dsRNA to control pests. (**A**) The chemical structure of the dendrimer nanocarrier and (**B**) The transdermal delivery system to enhance pesticidal effect of dsRNA. Reproduced with permission. (Adapted from Zheng Yang [33], with permission from the author).

A successful application of dsRNA delivery system to control pests was shown in Figure 2 [33]. Other representative applications of NCs-based delivery systems were listed in Table 1. The first successful dsRNA delivery system for pest control is based on a linear chitosan nanocarrier, dating back to 2010 [30,31]. Chitosan is polymer composes of β-(1–4) D glucosamine and N-acetyl-glucosamine that can be both biodegradable and commercialized production through a deacetylation of natural components of insect cuticle [34]. With both high delivery efficiency and non-toxicity, chitosan has been extensively studied in delivery systems and might be one of the most promising NCs to be applied in sustained agriculture [35]. By feeding pests with chitosan-encapsulated dsRNA, the chitinase gene of Anopheles gambiae (a main malaria vector) was efficiently silenced and larval lethality was observed [31]. Chitosan-delivered dsRNA has also been successfully applied to control other pests such as *Aedes aegypti* (a major vector for Zika, dengue fever, yellow fever, and chikungunya) and *Chilosuppressalis* (a destructive rice pest in China). 

Apart from chitosan, liposomes have been also employed as NCs to deliver dsRNA. As a mimic of amphiphilic cell membranes, liposomes are spherical vesicles composed of phospholipid bilayer with hydrophilic tails inward and hydrophobic heads outward. The structure not only provides a large cavity for presenting guest drugs at high densities, but also confer the ability to interact with hydrophobic cell membrane, which contributes to a penetration of biological barriers to reach their molecular targets [36]. Lin et al. employed a cationic liposome carrier to deliver oral dsRNA for control of the German cockroach *Blattella germanica* [37]. The dsRNA lipoplexes displayed a significantly slow degradation of dsRNA in the midgut of pest compared naked dsRNA, which resulted in enhanced effect of both gene silence and morality of *B. germanica*. 

Another widely used nanocarrier iscationic dendrimer [38]. Cationic dendrimer has a branched core-shell structure where hydrophobic core is around with hydrophilic repeating units to form a cationic sphere of different centric shells (each shell identified as one generation) [39]. The repeating units are commonly active functional groups that facilitate the surface functionalization to interact with various biological barriers such as degrading enzymes and cell membranes [40]. The physicochemical properties of dendrimers can meet multiple specific application requirements through a flexible change of core type, branching units, and surface functional groups [41]. This structure confers dendrimer thousands of cavities for drug loading as well as fast penetration into a broad range of tissue types [42]. The uptake of dendrimers by various tissues has been well demonstrated though in vivo experiments using fluorescent dendrimers. Yin and Shen et al. developed serious dendritic NCs based on perylenediimide derivatives (PDIs) which exerts extremely high fluorescence quantum yield for real-time tracking of NCs in situ [43]. Strong and persistent fluorescence of these NCs were detected in live tissues of various organisms such as model plants (*Arabidopsis thaliana*) and agricultural pests (*Drosophila melanogaster*, *Ostrinia furnacalis*, *Agrotis ypsilon*, *Aphis glycines*, etc.) [43,44]. These dendritic NCs were then applied to deliver dsRNA for pest control, resulting in largely enhanced efficacy of gene silencing and pest knockdown. Especially, the dendrimer was then employed in a transdermal dsRNA delivery system, which achieved a high gene silencing effect of 95.4% and satisfactory control of soybean pests. This efficient transdermal system established a foundation for further application of dsRNA sprays in field, offering a range of opportunities for a targeted pest control in smart agriculture [33]. Subsequently, the fluorescent dendrimer was converted to cheaper star-shaped NCs, by deleting the fluorescent core (PDIs) and simplifying synthesis procedure. The star-shaped NCs maintained all the advantages of previous dendrimer except fluorescent tracing [45]. All the studies above offer various practical routes to achieve a targeted and economic control of pests by specific dsRNA and its delivery systems. 

### 2.3. Sustained-Release Delivery Systems for Prolonged Duration of Pesticides

**Figure 3:**
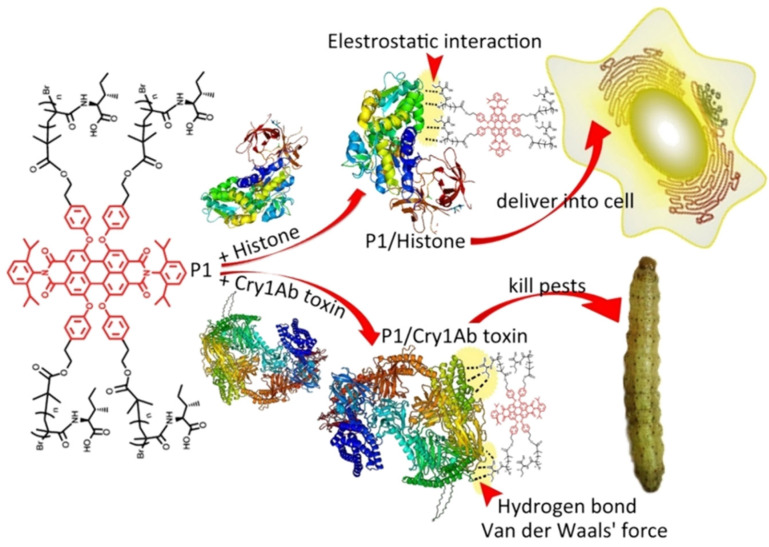
Schematic diagram of NCs-mediated delivery of *Bt* toxins for an effective control of unsensitive pests. Reproduced with permission. Adapted from Zheng Yang [54], with permission from the author.

Another advantage of introducing NCs in pesticides is sustainable release of AIs to achieve a prolonged duration of pesticides. Several successful applications are shown in Table 2. Among them, one of the most strategies is introducing NCs to deliver bioagents (e.g., pest-host pathogens, parasitoids, or their metabolic product with pesticide activity). An endotoxin-producing bacterium called *Bacillus thuringiensi* (*Bt*) is a widely used bioagents. The *Bt* endotoxin can effectively poison targeted pests while ensure safety to nontargets, through a special toxin-activated mechanism that only happened in special intestinal conditions of pests [52]. However, like dsRNA biological molecular, it also has unstable control effect under harsh environment. Maghsoudi, S. et al. developed a UV-blocking delivery system for *Bt* using Graphene oxide (GO) and olive oil. Grapheme and its derivatives GO offer ultrahigh surface areas with a single or a few layers of sp2 hybrid carbon atoms, making them ideal platforms for highly efficient AIs loading [53]. Olive oils have an UV absorbance and it is still remained to be utilized before becoming a suitable UV-protectant. The adding of GO into olive oils achieved a exclusively enhanced UV protection of olive oils from 40% to 90%. And this combined protective composite matrix composing of GO/oils further contributed to high viabilities of *Bt* after 96 h, leading high mortality (68.89%) of *Ephestia kuehniella* larvae (a worldwide pest of stored grains). This work provides a novel approach to enhance UV protection of pesticides with additive effect of both NCs and other traditional protectants. Another study applied dendritic NCs to develop an oral delivery system for delivery of *Bt* toxins, resulting in a high mortality of *A. ypsilon* (a pest non-sensitive to *Bt*) (Figure 3) [54]. 

The essential oils delivery system based on NCs is another successful application. As a promising biocontrol sources, the plant essential oils also have a high pesticidal activity, a low mammalian toxicity but a strong volatility, leading to a lack of persistence [67]. Two or more carefully timed applications may be required to ensure satisfactory management of pests. The disadvantage of essential oils has been changed after encapsulating them into nanoformulations. An antibiotic plant essential oils from *Eucalyptus citriodora* were made into nanoliposomes to fight against *Staphylococcus aureus* [55]. This antibacterial nanoformulation displayed high efficiency and long-term availability. Zeinab Ahmadi et al. reported a chitosan-mediated nanoencapsulation of essential oils (EOs) (extracted from *Achillea millefolium* L.) [16]. This EOs nanoformulation achieved a persistent release of AIs and prolonged acaricidal effect to a plant-feeding mite *Tetranychus urticae* Koch in fumigant experiments and contact lethality tests. 

A continuous release of Emamectin-benzoate over 200 h was also observed after an encapsulating it into microspheres composed of polymeric stabilizer polyvinyl alcohol and non-ionic surfactant polyoxyethylene castor oils. The microsphere also had excellent antiphotolysis performance and good leaf distribution [61]. Theses study demonstrated the feasibility of employing NCs as efficacy enhancers for bioagents that are unstable under harsh condition. Various NCs such as chitosan, liposomes, polymeric NCs, or mesoporous silica are used to deliver a multitude of biological AIs, including botanical pesticide PONNEEM, insecticidal deltamethrin and garlic oils [56,62,63,68]. 

Chemical AIs with unsatisfying formulation undergo a premature emission into the environment, resulting in short-lived control effects of pests and a toxic risk to nontargets. These disadvantages have been reversed through a NCs-based bifunctional delivery systems that offer protection and slow release of AIs. In these systems, mesoporous nanomaterials (MNs) such as nanoclay, activated carbon, and porous hollow silica are widely used as protective substrates [13]. They offer numerous well-ordered pores and easily modified surfaces for both drug loading and releasing. Also, high thermal and mechanical stability of MNs provide a potential for a persistent and high-quality release of AIs [69]. Natural montmorillonite modified by biopolymer chitosan have been employed to form mesoporous matrix for sustained release of herbicides. The effect of sustained release was only 27% release of herbicidal imazamox in the first 10 min, much lower than that of commercial formulations (86%) [59]. The encapsulation of abamectin into a self-prepared porous hollow silica NCs (PHSN) protected this pesticide from direct exposure to UV light and enabled a sustained release over 30 days [60]. These studies demonstrated the feasibility of employing NCs as efficacy enhancers for pesticidal AIs, through enhanced protection effect, increased leaf distribution, efficient delivery into pests and slow but persistent release. 

### 2.4. Stimuli-Responsive Systems for Controlled Release and Adjustable Degradation

As discussed above, more and more advanced NCs have been applied in delivery systems to enable pesticides with good protection, sustained release, low toxicity, and other excellent properties. In advanced smart systems, NCs was grafted with stimulation-responsive groups to facilitate a regulated release of AIs by stimulus conditions such as pH, thermal, light and enzyme of pests, which can not only control pests effectively but also reduce environmental contamination [70,71]. These systems have excellent response capability, which ensures pesticide release switches on for targets and off for nontargets. Representative stimuli-triggered systems for AIs and their corresponding functions were shown in Table 3. Enzyme-responsive systems have a potential of targeted control of pests due to the specificity of the enzyme-substrate interaction. Kaziem, A. E. et al. developed a delivery system for enzyme-responsive release of chlorantraniliprole based on hollow mesoporous silica NCs that were modified by α-cyclodextrin, contributing to a high larval mortality of the destructive crucifer-specialized pest, *Plutellaxylostella* [72]. Another enzyme-responsive system was constructed for delivery of emamectin-benzoate based on a copolymer matrix of silica epichlorohydrin carboxymethylcellulose. The pesticide was inserted into silica shells formed by TEOS using the emulsion polymerization method. Then the silica shells were modified using APTES for the formation of amino-functionalized silica microcapsules. Finally, the microcapsules were cross-linked with EMC to form the enzyme responsive microcapsules. Then the fabricated pesticides exerted a photo/thermal protective effect on insecticides, excellent cellulase stimuli-responsive properties, and finally achieved a sustained insecticidal efficacy against the significant aphid pest of peach trees, *Myzus persicae* [73]. 

The alkaline-response oral delivery systems play a role in targeted control of insect pests by triggering the release of insecticides in alkaline guts. Kumar et al. fabricated pH-dependent release system using two natural materials of alginate and chitosan, via an ionic pregelation and a polyelectrolyte complexation route [85]. The release of insecticidal acetamiprid was growing with the increase of pH from 4 to 10. A base-triggered release formulation of thiamethoxam was developed using biopolymeric clay hydrogels composites that had been synthesized, by crosslinking of carboxymethyl cellulose with citric acid in the presence of bentonite. In this system, a higher release of insecticides was observed at alkaline pH condition than neutral pH [86]. 

Recently, light-responsive delivery systems have gained considerable interest. A light-controlled delivery system was well developed by conjugating photolabile 2-nitrobenzyl with carboxymethyl chitosan to deliver herbicides (Diuron, a photosynthetic inhibitor) [87]. This system had a high photo-controlled release rate of 96.8% under solar stimulation while non-release without light exposure. The polydopamine (PDA) was capped with poly(N-isopropylacrylamide) (PNIPAm) to form a NIR-light/thermality triggered delivery system [62]. PDA was employed as a photothermal agent and PNIPAm acted as a thermosensitive gatekeeper and a pesticide reservoir. The PDA@PNIPAm nanocomposites exhibited well-defined core shell configuration and a photothermal response, which provided a foundation for targeted release of pesticides to selectively control diurnal pests. In another insecticide-delivery study, GO NCs was decorated with copper selenide compounds to form the delivery system that has both photothermal and photocatalytic performance. Photothermal property complemented with GO in delivery of chlorpyrifos to target pests. The photocatalytic property promoted the programmed degradation of pesticide residues in the offsite. This system achieved a good pesticidal effect (enhanced larval mortality above 35%) by targeted release and strong binding of pesticides to resist drift [79]. Another photocatalytic system based on TiO_2_ photocatalysts also contributed a photocatalytic oxidation degradation of abamectin pesticides after a temporal threshold for pest control [80]. These smart systems for degradation of pesticides provide a promising tool to reduce non-target accumulations.

## 3. Challenges and Perspective

The rapid development of nanotechnology brings new opportunities and driving forces for the agri-tech revolution [70]. Previous studies presented a series advanced nanodelivery systems for the existing AIs, which offered practical tools to achieve an efficient and sustainable pest management. First, the utilization of pesticide formulation improved water-solubility of hydrophobic AIs and enhanced stability. Second, nanodelivery system increase efficacy by protecting AIs against harsh condition and improving cell uptake of pesticides. Finally, eco-friendly delivery systems have reduced toxicity to nontargets by a controlled, smart release of AIs and a specific targeted control of pests. 

Despite all this progress, the current development of NCs-delivered pesticides is still at an early stage. There is insufficient information about their impact on human health and ecosystem safety. Both NCs-delivered pesticides and other pesticides at nanoscale (defied as nanopesticides) has been a growing concern for the public due to their toxicological risk assessment. Meanwhile, a unified assessment criterion is also needed before commercialization [1]. The European Food Safety Authority (EFSA) have made a guidance for risk assessment of nanopesticides in food and feed, suggesting all the accessory ingredient that contributes to final pesticidal effects should be concerned, including coformulants/excipients such as surfactants, solvents, carriers, and wetting agents [88]. Recently, two comprehensive frameworks for risk assessment of nanopesticides about human health or ecology have been reviewed [1,89]. The additional concerns and tests in risk assessment were suggested even all the ingredients of a certain nanoformulation was safe, because the nanopesticides with unique characteristics at nanoscale might have a distinct fate and behavior [1]. The evaluation of NCs toxicity still has difficulties and the toxicokinetics of NCs are also challenged [90]. Model predication on toxicokinetic might help us estimate toxic effects of NCs-delivered pesticides and other nanopesticides. Boxall, A. B. et al. provided a starting point for assessing risk of nanopesticides. They established a model that predicted the nanopesticides absorption in the soil earthworms, by incorporating the release rate and distribution of pesticides [89,91]. However, model predictions are complicated, as there are too many variations involved, such as nanopesticides (sizes and properties) and other variable factors from ecological or biological systems. For this reason, the ideal models and toxicity predictions in field or in organisms are still scarce. The experience gained in the area of nanomedicine might help characterize and assess nanopesticides [1]. In summary, much remains to be discovered in the world of nanopesticides risk assessment. 

Another strategy to reduced toxicity concern is using green materials or green synthesize for NCs production. As far as we know, the frequently reported NCs such as MOFs, adsorption resin, activated clay, activated alumina, and activated carbon have a nondegradable nature even though they have excellent delivery effects [13,92]. Nondegradable NCs exhibited long-term stability in during the drug delivery processes while showed toxicity to cells or environment, which is still a major issue to be resolved. The natural-derived polymers (such as starch) might be good alternatives due to their degradability, renewability, and low price. Efforts have been made to develop starch-based NCs to facilitate the delivery of pesticidal AIs, nutrients, and biostimulants into plant tissues [93]. However, there are still some drawbacks during the starch NCs producing process. The starch NCs are commonly produced by acid hydrolysis, which leads to long processing time and residual toxic waste that are harmful to the environment [94]. The conventional chemical synthesis process commonly involves toxic chemicals like protective agents to maintain the reaction stabile, which leads to enhanced toxicity to environment or nontargets. Relatively nontoxic materials or green synthesis methods is conducive to reduction of reaction time and increase a catalyst utilization by products.

Thus, it is necessary to use relatively nontoxic materials or green methods to synthesize or modify NCs. Biosynthesis, based on living organism or its extracts, is an important part of green technology. The waster cyanobacteria, with both biocompatibility and microalgae surface for drug-loading modification, have reported to be modified as NCs for delivery and controlled release of avermectin [95]. The biosynthesis of silver nanoparticles has been well documented using leaf extract (*Acalypha indica*), bacteria (psychrophilic bacteria), yeast (*Humicola* sp.), and fungi (*Aspergillus terreus*) [55,96,97,98,99,100]. These strategies provide new sight to minimize harmful chemical reactions/products during NCs synthesis. Beside biological synthesis, the physical methods such as ultrasonic synthesis provide novel insight for reduced waste products in chemical synthesis. The low frequency ultrasound (20 kHz) was found to be able to efficiently reduce the size of oil droplet in an aqueous suspension. Due to the de-agglomeration effect and the reduction of droplet size, ultrasonic processor could be promising source to generating nano-size dispersions in pesticidal formulations. A green synthesis of micro hydrogel composite was achieved by using renewable starch and cellusenanowhiskers. These green materials were then modified by ultrasound. Ultrasound contributes to disaggregate particles and prevent coalescence of this emulsion system. An enhanced controlled release performance of this green microhydrogel was achieved according to kinetic results. 

**Figure 4:**
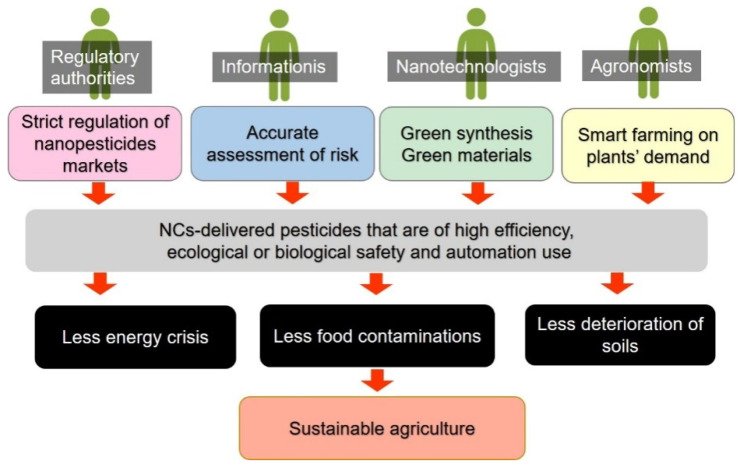
A possible future work of NCs-based pesticides and the potential future of agriculture.

All the processes of physical, chemical as well as biological methods are being developed to meet certain requirements of NCs synthesis. Chemical synthesis is most effective means to produce NCs and has the most potential to be scaled up for large-scale production. The development of biosynthesis and utilization of physical methods could be alternatives for chemical methods, in an eco-friendly manner and thus reducing the reliance on the toxic chemicals. However, each of the existing protocols suffers from their intrinsic defects. Thus, it is still an open challenge to combine all the methods to produce NCs with less side effect on an industrial scale. A possible future work is presented in Figure 4, which might be associated with a deep cooperation of specialists in various relevant fields. 

## 4. Conclusions

NCs-delivered pesticides are giving a fresh insight into sustained agriculture for a greater control efficacy and fewer side effects. It is increasing clear that the smart nanodelivery systems developed in past, present and further will plays vital role in dealing with the worldwide issues like the energy crisis, food contaminations, and deterioration of soils. Solving these issues will require strong collaboration among agronomists, nanotechnologists and informationist, to develop and apply multi-functional NCs-delivered pesticides that are beyond the properties of targeted control and regulated release. The responsibility of farmers could be to find the right time to degrade a smart pesticide by a balance between the high effect and less residues.

## Figures and Tables

**Table 1:** Successful application of nanodelivery system for gene silencing and targeted control of pests.

NCs	Pest	Improved Properties and Pest Control Effects	Reference
Chitosan	*A. gambiae*	Two key genes, *AgCHS1* and *AgCHS2*, can be repressed by chitosan/*AgCHS* dsRNA-based nanoparticles through larval feeding	[31]
Chitosan	*A. aegypti*	Reduced expression level of targeted *AaSNF7* (38%) and *AaSRC* (90%) through oral delivery of dsRNA	[32]
Chitosan	*C. suppressalis*	57% reduction of glyceraldehyde 3 phosphate dehydrogenase gene in whole body and 92% mortality of larvae.	[46]
Chitosan	*A. aegypti*	Enhanced gene silencing effect (20–65%) depending on the targeted genes (*IAP*, *SNF7*, *SSK*, and *OTK*) and increased larvae mortality	[47]
liposomes	*B. germanica*	Slowing down the degradation of dsRNA in midgut, significantly inhibiting of α-tubulin expression and increased mortality of pests	[37]
Carbon quantum dot	*A. glycines*	Enhancing gene silencing effect by the delivery of aerosolized siRNA-NCs, increased reduction of aphid body mass.	[48]
Carbon quantum dot	*A. aegypti*	Reduced expression level of targeted *AaSNF7* (60%) and *AaSRC* (29%) through feeding CQD/dsRNA; mortality of pests was 38% and 32% for dsAaSRC and dsAaSNF7	[32]
Dendrimer	*Heliothis* *armigera*	Efficiently enter into various larval tissues and enhanced larvae mortality (83.3%)	[49]
Dendrimer	*A. glycines*	Efficiently knockdown of targeted genes (effect ranging from 86.86 to 58.87%) and high mortality up to 81.67%	[50]
Dendrimer	*O. furnacalis*	Efficient inhibition of targeted gene by oral delivery and severe defects in larval growth and molt leading to death	[43]
Dendrimer	*D. melanogaster*	Efficiently knock down of multiple genes involved in DDT resistance and enhanced sensitivity to DDT	[51]

**Table 2:** Successful applications of using nanodelivery systems for slow, sustainable release of AIs.

NCs	AIs	Pest	Improved Capability and Pest Control Effects	Reference
Nanoliposomes	EOs from *Eucalyptus citriodora*	*S. aureus*	High efficiency and long-term availability of antibiotics	[55]
Nanolipid (corn oil, beeswax)	Insecticidal deltamethrin	*-*	Controlled release and increased photo-protection	[56]
Liposomes	Antimicrobial peptides	Foodborne pathogens	Enhanced antibacterial activity against Listeria monocytogenes and Escherichia coli	[57]
Polyethylene glycol	EOs from garlic	*T. castaneum*	Decreased volatilization of essential oils, retained 80% pest control efficacy over 5 months	[58]
Chitosan	EOs from *Achillea millefolium* L.	*T. urticae Koch*	A persistent release of AIs and prolonged acaricidal effect	[16]
Montmorillonite and chitosan	Herbicidal imazamox	Weeds	Only 27% release of herbicidal imazamox in the first 10 min, much lower than that of commercial formulations (86%)	[59]
Hollow silica NPs	Avermectin	*-*	remarkable UV-shielding for pesticides, a sustained release of pesticides over 30 days	[60]
Composite microspheres	Emamectin -benzoate	*-*	Excellent anti-photolysis and good leaf distribution, controlled release properties,	[61]
CSNs-TPP- PONNEEM ^1^	Botanical PONNEEM (R)	*H. armigera*	88.5% antifeedant activity and 90.2% larvicidal activity against pests	[62]
CH-BSLNs ^2^	Insecticidal deltamethrin	*-*	74.5% of deltamethrin remained after exposure to UV irradiation for 2 h	[63]
Alpha-pinene, linalool and silica	Insecticidal terpenes	*S. litura.* and *A. janata* L.	Enhanced the antifeedant against insects and longer shelf-life of pesticides	[64]
Biogenic silica	Theneem extract	*A. crassispinus* *ant specie*	Improving stability of pesticides to four-fold and sequential release profiles of pesticides	[65]
Copolymer (styrene and methacrylic acid)	Insecticidal avermectin	*-*	Excellent storage stability, improved resistance to ultraviolet light, sustained release and increased retention ratio on foliage	[20]
GO/olive oil	*B. thuringiensis*	*E. kuehniella*	Highest viability (50.62%) after exposure to for 96 h	[53]
Hybrid hydrogels (biosorbents and sodium alginate)	Botanical azadirachtin	-	Enhanced resistance against simulated sunlight	[66]

^1^ CSNs-TPP-PONNEEM, chitosan nanoparticles that were prepared by cross linking agents’ glutaraldehyde and tripolyphosphate. ^2^ CH-BSLNs, chitosan-coated beeswax solid lipid nanoparticles.

**Table 3:** Stimuli-responsive nanosystems and their corresponding functions.

Nanocarriers	Pesticide	Stimuli	Improved Capability and Pest Control Effects	Reference
Hollow mesoporous silica	Chlorantraniliprole	Pest enzyme (α-amylase)	High larval mortality of *P. xylostella*	[74]
Copolymer matrix	Emamectin benzoate	Pest enzyme (cellulase)	Cellulase stimuli-responsive properties and sustained control of *M. persicae*	[75]
Silica-IPTS-PE ^1^	Pendimethalin	Urease	Enhanced thermal and light stability of pendimethalin, increased duration and strengthened herbicidal activity	[65]
Natural alginate and chitosan	Insecticidal acetamiprid	Alkaline (pH of pest intestine)	Higher release of insecticides at alkaline pH condition than neutral pH	[76]
Clay hydrogels	Insecticidal thiamethoxam	Alkaline (pH of pest intestine)	Enhanced release of pesticides in alkaline pH condition	[19]
2-nitrobenzyl carboxymethyl chitosan	Herbicidal diuron	Photo	High photo-controlled release rate of 96.8% while non-release without light exposure	[77]
PDA-PNIPAm ^2^	Insecticidal imidacloprid	Photo	Good photothermal response capability, potential in selectively control of diurnal pests	[78]
GO NCs	Chlorpyrifos	Photo	Photothermal and photocatalytic performance, programmed pesticide residue degradation, resistance to drift, enhanced larval mortality (>35%)	[79]
TiO_2_ NCs	Abamectin	Photo	photocatalytic oxidation degradation of pesticides	[80]
Perylene-3-ylmethanol	Herbicidal 2,4-D ^3^	Photo	Increased cell uptake of pesticides in plant, improved herbicidal activity, efficient photo-regulated release	[81]
Mesoporous silica	Herbicidal 2,4-D	temperature, pH and ionic strength	A controlled release pattern of pesticides, decreased soil leaching of 2, 4-D sodium salt	[82]
Salicylaldehyde modified mesoporous silica	Chlorpyrifos	pH	Decreased release of pesticides with the pH increasing	[83]
Alginate-grafted anisotropic silica	λ-cyhalothrin	pH	Decreased release of pesticides in the emulsion from 99.7% to 13.5%	[84]

^1^ Silica-IPTS-PE, isocyanate-functionalized silica cross-linked with polyethylenimine. ^2^ PDA-PNIPAm, polydopamine capped with poly(N-isopropylacrylamide). ^3^ 2,4-Dichlorophenoxyacetic acid (2,4-D).
